# Dual Implementation of Phrenic and Hypoglossal Nerve Stimulators in a Patient With Obstructive and Central Sleep Apnea and Associated Cardiac Arrhythmias

**DOI:** 10.7759/cureus.104777

**Published:** 2026-03-06

**Authors:** Arindam Ghosh, William Anderson, Elizabeth Rivera, Laurie Joughin, Abhay Sharma

**Affiliations:** 1 Medicine, University of South Florida Morsani College of Medicine, Tampa, USA; 2 Sleep Medicine, University of South Florida Morsani College of Medicine, Tampa, USA; 3 Sleep Medicine, Tampa General Hospital, Tampa, USA; 4 Otolaryngology - Head and Neck Surgery, University of South Florida Morsani College of Medicine, Tampa, USA

**Keywords:** central sleep apnea, hypoglossal nerve stimulator, mixed sleep apnea, obstructive sleep apnea, phrenic nerve stimulator

## Abstract

When patients with obstructive sleep apnea (OSA) and central sleep apnea (CSA) fail to benefit from first-line therapies, combinations of new treatment modalities may be beneficial. The hypoglossal nerve stimulator (HNS) and phrenic nerve stimulator (PNS) are two exciting options in the management of OSA and CSA, respectively. A 72-year-old man with concurrent CSA and OSA underwent sequential implantation of a PNS followed by a unilateral HNS, and his response to each device was monitored with polysomnography (PSG). This patient also had a significant history of atrial flutter and premature ventricular contractions refractory to ablation, which his cardiologist had attributed to his sleep apnea. Implantation of the PNS device diminished the features of CSA on follow-up PSG. While his Apnea-Hypopnea Index (AHI) improved from 66 events/h to 30 events/h, the obstructive component of his sleep apnea remained elevated. Continued obstructive events and daytime sleepiness prompted implantation of the HNS. A follow-up PSG revealed an AHI of 12 events/h and significant improvement in nighttime oxygenation. Thus, simultaneously reducing upper airway collapse and central ventilatory instability may confer greater improvement than either approach used alone. Utilizing hypoglossal and phrenic nerve stimulator devices in unison may be an efficacious approach in the management of complex cases of sleep apnea.

## Introduction

Central sleep apnea (CSA) and obstructive sleep apnea (OSA) often occur concurrently. CSA presents with pauses in breathing with an open upper airway. Congestive heart failure (CHF) is one of the best-established mediators of this disease [1,2]. Neurologic disorders, narcotic use, and living at high altitudes also factor into CSA pathogenesis, and CSA is far less common than OSA. CSA stems from excessive reactivity from the brainstem that results in a cycle of hyperventilation and central apnea, a concept known as high loop gain [3,4]. OSA is characterized by repetitive cycles of upper airway collapse, causing arousals from sleep or drops in oxygen saturation. A variety of anatomic and physiologic factors contribute to OSA, which is highly prevalent in the general population [5]. While treatment with different varieties of positive airway pressure (PAP) therapies is highly effective, compliance is commonly an issue [6]. In the last decade, new options have emerged for treating sleep-disordered breathing. Hypoglossal nerve stimulation (HNS) is one promising modality in the management of patients with predominantly OSA that is refractory to continuous PAP (CPAP) [7,8]. The most commonly used device in the current landscape (Inspire, Inspire Medical Systems, Golden Valley, MN, USA) is comprised of three components: a respiratory sensor, a pulse generator, and a cuff electrode that wraps around the hypoglossal nerve. The sensor detects inspiration and signals to the electrode to contract muscles in the tongue and limit obstruction of the airway. Adverse effects include tongue movement restrictions or atrophy [8]. Clinical trials, such as the STAR trial, suggest this device’s long-term efficacy [9,10]. CSA can also be treated with alternatives to PAP therapy. One of these alternatives is a phrenic nerve stimulator (PNS), which caters to patients with predominantly central events. PNS sits in a vein running parallel next to the phrenic nerve in the upper chest to generate a physiologic breathing pattern. In the Pivotal trial, the now FDA-approved device (Remede, Zoll Respicardia, Inc., Minnetonka, MN, USA) was shown to improve AHI, oxygen desaturation index, and central apnea index through 36 months. Documented complications include lead displacement and sensory change in an area distinct from the diaphragm [11].

The underlying reasons for patients concurrently presenting with CSA and OSA vary. Since both CSA and OSA are significant causes for health concern, addressing both is paramount. To date, there has been no vetted framework for identifying suitable candidates for dual HNS and PNS implantation. Here, we present a patient with predominantly CSA related to cardiovascular comorbidities, who was treated first with PNS and subsequently underwent implantation of an HNS. Records were evaluated retrospectively. Multiple sleep studies were obtained that were a combination of PSG and respiratory polygraphy, depending on the time point along the treatment pathway.

## Case presentation

A 72-year-old man with postural orthostatic tachycardia syndrome and suspected Ehlers-Danlos syndrome presented with features of both OSA and CSA. He had a body mass index of 22, hypertension, hyperlipidemia, atrial flutter, and premature ventricular contractions (PVCs) refractory to multiple attempts at ablation. His cardiologist had strongly urged him to seek treatment for his sleep apnea to resolve his PVCs.

Seven years prior, his central sleep apnea failed to respond to CPAP and bilevel PAP (BiPAP). He was unable to tolerate adaptive servoventilation (ASV), stating that it disrupted his sleep and the pressure was excessive. He did not seek additional care for his sleep apnea until three years ago. At this time, he reported nocturnal awakenings every 1-2 hours. Although he denied being aware of snoring or choking, his wife witnessed apneas. In the morning, he would awake with dyspnea, exhaustion, and headaches. He would often doze off during the daytime as well. Efforts to improve his sleep hygiene, such as by reducing daytime naps along with avoiding food and screentime immediately before bedtime, did not tangibly improve his symptoms. Weight loss and trial of a mandibular advancement device (MAD) failed to produce any benefit, and he stopped using the MAD as a result. Diagnostic PSG at this time without a MAD showed an Apnea-Hypopnea Index (AHI) of 65.9. He experienced 94 obstructive, 103 central, 23 mixed, and 58 hypopnea events. His central apnea index was 24.4, and his obstructive apnea index was 22.3. Cheyne-Stokes respirations were present. His O2 saturation nadir was 68%, and his oxygen desaturation index (ODI) was 63.8. His time with blood oxygen saturation below 90% (T90) was 11.7 minutes. AASM version 2.1 was used to score the sleep study. During this sleep study, the heart rhythm was a normal sinus rhythm with frequent PVCs.

Ten months following baseline PSG, he underwent implantation of a PNS to treat his CSA, given it was the predominant disease. Ten weeks following PNS device activation, he reported fewer awakenings and improved daytime functioning. Follow-up PSG suggested resolution of CSA and Cheyne-Stokes respiration with PNS set between 2.6 and 3.0 V. Nineteen months after baseline PSG, his subsequent PSG revealed an AHI of 30 events/h, with the majority being obstructive. Consistent with his prior presentation, there was a significant positional component to his AHI, with his supine AHI of 51 events/h and non-supine AHI of 2 events/h. Per hour, he experienced 18.3 obstructive apneas, 3.1 central apneas, 1.2 mixed apneas, and 7.2 hypopnea events. He cited being unable to sleep on his side for extended periods due to upper shoulder and back pain, which had been aggravated in part by the PNS system. His average oxygen saturation was 92%, and his time spent with blood oxygen saturation below 88% (T88) was 31 minutes. His SpO2 nadir was 78%. His ODI was 25.8. AASM version 2.5 was used to score the sleep study. During this sleep study, the heart rhythm was a normal sinus rhythm with occasional PVCs. An electrocardiogram around this timeframe continued to show premature atrial and ventricular beats.

**Figure 1:**
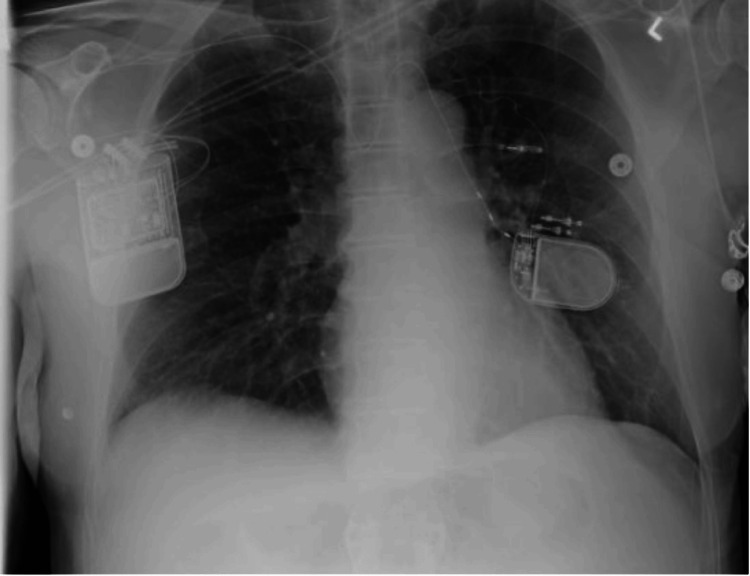
Chest X-ray showing Inspire and Remede devices The chest X-ray illustrates the hypoglossal nerve stimulator (HNS) device implanted in the right hemithorax and the phrenic nerve stimulator (PNS) implanted in the left hemithorax.

Given his continued symptoms, HNS was considered a viable option. A drug-induced sleep endoscopy confirmed no complete concentric collapse, and he underwent implantation of the HNS device roughly 2.5 years after baseline PSG. When fully activated, the device had the following settings: 1.7 V, pulse width 90, and pulse rate 30. A follow-up portable home sleep study, which was roughly three years after his initial study, revealed that his overall AHI had improved to 12.4. His average oxygen saturation was 99.8%. His T88 was 2.3 minutes, and his SpO2 nadir was 84%. His ODI was 4.9. Moreover, the Inspire device had improved supine AHI improved to 21.2 events/h, while non-supine AHI was 1.1 events per hour. AASM version 2.5 was used to score the sleep study. Per hour, he experienced 10.7 obstructive apneas, 0.5 central apneas, 0 mixed apneas, and 1.2 hypopnea events. Electrocardiograms from his cardiologist in the two years following HNS implantation failed to identify an arrhythmia. Figure 1 illustrates this patient's properly positioned HNS and PNS. Table 1 compares polysomnography data across the three study dates.

**Table 1:** Polysomnography metrics at baseline and following therapy Patient’s PSG data at baseline, with only phrenic nerve stimulator (PNS) implantation, and with both phrenic nerve stimulator (PNS) and hypoglossal nerve stimulator (HNS) in place. *The polysomnography laboratory updated its software to a version that recorded T88 only between the first study and the subsequent ones.

	Baseline	PNS only	HNS and PNS
Apnea-Hypopnea Index (AHI)	65.9	29.7	12.4
Obstructive apnea index	22.3	18.3	10.7
Central apnea index	24.4	3.1	0.5
O2 saturation nadir	68%	78%	84%
Oxygen desaturation index (ODI)	63.8	25.8	4.9
Epworth sleepiness scale	13	11	12
O2 desaturation time^*^	T90: 11.7 min	T88: 31.5 min	T88: 2.3 min

## Discussion

This case study highlights the potential of a tandem of Inspire and Remede in the management of combined refractory OSA and CSA. Similar success has been recently reported in the joint use of PNS and HNS stimulators [12]. Unlike the prior case, however, this patient had a significant history of cardiac arrhythmias related to his sleep apnea, signifying a more detrimental disease course, and there were some indications of improvement based on electrocardiograms and sleep study heart monitoring. Moreover, the prior case was limited to home-sleep tests rather than in-lab PSGs. For complex cases, such as those with mixed CSA and OSA, the American Academy of Sleep Medicine recommends PSG over home sleep assessment for more accurate differentiation of sleep-disordered breathing subtypes [13]. 

The order of implantation of the device can vary based on the pathophysiology of the sleep-disordered breathing. This is important to consider, given the requisites for insurance approval for each device. In our case, the patient was known to have an obstructive component, and the central component was more prominent, permitting implantation of the PNS. Criteria for PNS implantation are a central apnea/hypopnea index above 15 events/h or an AHI over 15 events/h, with a central apnea index greater than the obstructive apnea index. HNS therapy criteria include having an AHI over 15 events/h along with a central and mixed index less than 25% of the events. Once the central events have been well treated, treatment of HNS could then be considered in this patient.

## Conclusions

This patient’s clinical course highlights how a case of refractory mixed obstructive CSA and OSA can be managed with the combined use of an HNS and PNS. Concurrently addressing upper airway collapse and aberrant central respiratory drive can significantly ameliorate sleep-disordered breathing, as indicated by this patient’s improvement in AHI and measures of desaturation. Given the large population of patients with this disorder and the continued need for PAP alternatives, this scenario will likely become more prevalent in sleep clinics as the therapies grow in usage. Potential criteria for dual HNS and PNS implantation may include elevated AHI with significant obstructive and central events, along with failure with CPAP and ASV. Conclusions from this case report may shape new hypotheses regarding the utility of dual implementation in mixed sleep apnea, and formulating criteria for identifying optimal candidates may significantly improve patient care.
